# Genome-wide DNA methylation study identifies significant epigenomic changes associated with internalized stigma in adults with non-specific chronic low back pain

**DOI:** 10.3389/fpain.2022.1021963

**Published:** 2022-11-28

**Authors:** Edwin N. Aroke, Joanna M. Hobson, Travis Ptacek, Pamela Jackson, Burel R. Goodin

**Affiliations:** ^1^ School of Nursing, University of Alabama at Birmingham, Birmingham, AL, United States; ^2^ Biobehavioral Pain Lab, Department of Psychology, University of Alabama at Birmingham, Birmingham, AL, United States; ^3^ Center for Clinical and Translational Science, University of Alabama at Birmingham, Birmingham, AL, United States; ^4^ Center for Addiction and Pain Prevention and Intervention (CAPPI), University of Alabama at Birmingham, Birmingham, AL, United States

**Keywords:** chronic low back pain (CLBP), internalized stigma, RRBS - reduced representation bisulfite sequencing, DNA methylation, hippo signaling, nonspecific chronic low back pain

## Abstract

Non-specific chronic low back pain (cLBP) represents a common musculoskeletal condition with no identifiable cause. It cannot be diagnosed with conventional neuroimaging techniques such as computerized tomography (CT). The diagnostic uncertainty that characterizes non-specific cLBP can lead to stigmatizing responses from others that can become internalized Among individuals with non-specific cLBP, internalized stigma is associated with greater pain intensity and disability. Yet, no study has examined the biological mechanism linking high internalized stigma to worse outcomes in individuals with non-specific cLBP. We aimed to identify differentially methylated loci (DML), enrichment pathways, and associated network interactions among individuals with non-specific cLBP experiencing low vs. high internalized stigma. We examined DNA methylation in whole blood samples from 48 adults, ages 19–85, using reduced representation bisulfite sequencing (RRBS). After controlling for age, sex, race, and multiple testing, differentially methylated loci (DML) differed in adults with low vs. high internalized stigma by at least 10% and *q* < 0.01 in 3,665 CpG sites: 2,280 hypomethylated and 1,385 hypermethylated. Gene ontology (GO) analyses of the annotated genes from these sites revealed significant enrichment of 274 biological processes, 29 cellular components, and 24 molecular functions (adjusted *p* < 0.05). The top enriched molecular functions regulate protein binding and DNA binding of transcription factor activity. Pathway analyses indicated that many functional genomic pathways, including Hippo Signaling, Melanogenesis, and Pathways in Cancer, were enriched with differentially methylated genes. Also, there was a significant interaction between relevance pathways such as *P53*, *mTOR*, *PI3K-Akt*, and *Wnt signaling* pathways. These pathways have previously been associated with neuroinflammation, neurodegeneration, and stress-related conditions. Thus, findings point to possible stress-induced DNAm changes as the link between high levels of internalized stigma and worse outcomes in adults with non-specific cLBP.

## Introduction

For years, non-specific chronic low back pain (cLBP) has been a leading contributor to years lived with disability worldwide, representing about 90% of all cLBP cases (1–3). Non-specific cLBP is associated with increased health care utilization, escalating health costs, years lived with disability, decreased productivity, and overall decreased quality of life (1). By definition, non-specific cLBP is “a back pain problem that has persisted for at least 3 months and has resulted in pain on at least half the days in the past 6 months” with no evidence of an underlying pathological abnormality (4). Therefore, non-specific cLBP cannot be diagnosed with conventional neuroimaging techniques such as computerized tomography (CT) scan (3). Because individuals with non-specific cLBP often have no identifiable cause, it can result in mistrust, stigma, and stress as the individuals with non-specific cLBP navigate the health care system.

Stigmatization refers to the devaluing, invalidating, and discrediting of the experiences of individuals that deviate from societal norms (5, 6). Stigma towards individuals with non-specific cLBP can be reflected in different ways, including anxiety and negative stereotypes from experiencing pain without a clear pathoanatomical condition. Stigmatized individuals often show greater pain catastrophizing, worse depressive symptoms, poorer physical outcomes, greater psychological distress, and a sense of injustice, but one of the worst consequences is the internalization of stigma (5, 7). According to the stage model of self-stigma (i.e., internalized stigma), others' stigmatizing behavior will be detrimental only when the stigmatized individual is aware of, agrees with, and applies the stigmatizing attitudes to the self (8, 9). In other words, internalized stigma is a form of stigma that involves applying the negative stereotypes, attitudes, and beliefs to yourself.

Chronic pain patients with internalized stigma report a sense of worthlessness and feelings that “others don’t believe that [their] pain is real and attribute [their] pain to drug seeking, laziness, or mental problems” (10). Studies have shown that experiences of internalized stigma are associated with symptoms of insomnia, depressive symptoms, and pain symptoms in vulnerable populations (11, 12), partly because people conceal stigma (10). Internalized stigma and the associated chronic pain-related stress induce psycho-neuroendocrine responses that can alter an individual's perception of pain (13). Specifically, chronic stress dysregulates the hypothalamo-pituitary adrenal axis, and alter inflammatory processes in many chronic pain conditions (14–16). In fact, cLBP has been conceptualized as stress-related “wear and tear” or allostatic overload that results from maladaptation to environmental challenges (17, 18). We recently found that chronic stress predicts non-specific cLBP severity, and greater chronic pain stigma positively correlated with perceived injustice, which in turn correlated with greater non-specific cLBP severity (19). Yet, the biological mechanism by which high levels of internalized stigma induces these changes and predisposes individuals to worse cLBP remains relatively unknown.

Epigenetic modifications provide a dynamic mechanism through which lived experiences, such as chronic stress, exert long-term effects on gene expression, biological pathways, and downstream phenotypes without altering the DNA sequence (20). Decades of research has shown that chronic stress and stress hormones can induce epigenetic changes (e.g., DNA methylation, DNAm) in the brain, altering gene expression and maintaining long-lasting neuroplastic changes (21). DNAm involves the enzymatic addition of a methyl group to the 5th carbon of cytosine nucleotides when a cytosine-guanine (CpG) dinucleotide is present. Catalyzed by DNA methyltransferases, methylated cytosine can alter DNA structure, allowing differential gene expression by regulating transcription factor binding, methyl-binding protein recruitment, or chromatin remodeling (20, 22). These heritable processes are tissue specific and play an essential role in physiological and pathophysiological processes (22). Growing evidence correlate differential DNAm of genes involved in stress regulation and neuro-inflammatory processes with non-specific cLBP (23–25). However, evidence of the relationship between internalized stigma and DNAm changes in individuals with non-specific cLBP is lacking so far.

We hypothesized that internalized chronic pain stigma positively correlates with perceived stress and is associated with DNA methylation alterations in stress regulatory pathways in adults with non-specific cLBP. To test this hypothesis, first, we determined the correlation between internalized stigma and perceived stress in individuals with non-specific cLBP. Then, we examined DNAm profiles in individuals with low vs. high internalized stigma. Finally, we investigated gene ontologies (GO) and functional genomic pathways enriched by genes annotated to differentially methylated loci between individuals with low vs. high internalized stigma.

## Methods

### Design and samples

The current study analyzed data from a larger ongoing cross-sectional study titled Examining Racial And SocioEconomic Disparities in cLBP (ERASED) study (R01MD010441). The samples included in this manuscript were collected between November 2018 and November 2019. Forty-eight individuals (21 males and 27 females), ages 19–85, with non-specific cLBP were recruited to participate. Following an initial telephone screening to determine eligibility for the study, the electronic medical records of all participants were reviewed and diagnosis of non-specific cLBP confirmed using the joint clinical practice guidelines from the American College of Physicians and the American Pain Society (26). This medical history review was used to validate self-reported diagnosis of non-specific cLBP. Individuals were included in the study if low back pain had reportedly persisted for at least three consecutive months and was present for at least half the days in the past six months (27).

We have previously published details of the recruitment strategy, inclusion, and exclusion criteria (19, 28). Briefly, participants were excluded if they had any conditions that could confound the interpretation of results, including cLBP attributable to infection, trauma, malignancy, or ankylosing spondylitis, systemic infection, chronic inflammatory disease, poorly controlled diabetes, systemic rheumatic disease (e.g., rheumatoid arthritis, systemic lupus, erythematosus, fibromyalgia), and neurological diseases. Of note, participants self-identified their assigned sex at birth (male, female, or intersex) and “race” (American Indian or Alaska Native, Asian, Black/African American, Native Hawaiian or Other Pacific Islander, and White). Several recruitment strategies were used to ensure diversity in the sample, with special attention to recruiting an equal sample of White and Black Americans of similar age and living with non-specific cLBP. Additionally, we posted flyers around the University of Alabama at Birmingham (UAB) community and pain clinic to recruit participants of varied socioeconomic statuses.

This study was approved by the institutional review board (IRB) at the UAB and is abiding by all the standards of the Declaration of Helsinki. Written informed consent was obtained from all participants prior to their inclusion in the study. Responses to study questionnaires and venous blood samples were collected in accordance with the guidelines and approval from the IRB. Also, this study was conducted in accordance with the cLBP research standards put forth by the Research Task Force of the National Institutes of Health Pain Consortium (4).

### Measures

After enrollment in the parent study, all participants completed an initial visit in which informed consent, demographic, and self-reported measures were collected. To understand the relationship between DNAm, stress, and stigma among adults with non-specific cLBP, as well as recognize the heterogeneity of these outcomes within this community, participants were asked to complete self-report measures on experiences of chronic pain stigma and perceived stress related to non-specific cLBP. For multifaceted assessment of the participants' phenotypes, we used the following three well-established scales:

#### Graded chronic pain scale (GCPS)

The GCPS is a well-validated and widely used self-reported instrument that assesses two dimensions of chronic pain: pain intensity and pain-related disability. It is composed of 7 items that score pain intensity (0–10) and pain-related disability (0–10) over the past 6 months. On the pain intensity measure, 0 means no pain and 10 means the most severe pain over the past 6 month. Similarly, 0 means no pain related disability and 10 means the worst pain-related disability, in the past 6 months. Higher GCPS scores represent higher pain intensity and greater pain-related physical disability. The GCPS is widely used scale with a good to excellent internal consistency reliability among individuals with cLBP (29, 30) In our study sample, GCPS has a robust internal consistency reliability (Cronbach *α* = 0.82).

#### Perceived stress scale (PSS)

The PSS is well validated and reliable self-reported 10 items questionnaire that measures the participants' psychological stress over the last month. Participants’ scores range from 0 to 40, with lower scores indicating lower levels of perceived stress. PSS has an acceptable to excellent internal consistency reliability (Cronbach's *α *= > 0.84) (31). In our study sample, PSS has an acceptable internal consistency reliability (Cronbach's *α *= 0.85).

#### Internalized stigma of chronic pain (ISCP)

The ISCP assesses the internalization or absorption of negative attitudes about chronic pain. This self-reported instrument is composed of 21 items that assess five dimensions of internalized stigma: alienation, stereotype endorsement, discrimination experience, social withdrawal, and stigma resistance (13). Stigma resistance items were reverse coded before inclusion in the total score, and higher ISCP scores reflect greater experiences of chronic pain stigma. The ISCP scale is a widely used, valid, and reliable instrument (13) with an excellent internal consistency in our sample (Cronbach's *α* = 0.91). ISCP scores range from 1 to 4 and we categorized ISCP into low stigma (ISCP < 2) vs. high stigma (ISCP ≥ 2). The cutoff of 2 was used to capture clinically relevant levels of stigma, and *Methylkit* that is used for DNAm analyses requires a dichotomous dependent variable (case-control).

### Genomic DNA extraction and sequencing

Blood was drawn from a vein in the participant's arm into a tube containing ethylene-diamine-tetra-acetic acid (EDTA). The EDTA tube was centrifuged at 3,000 rpm for 10 min, and the buffy coat was carefully extracted and stored at −80 degrees until ready for genomic DNA extraction. Genomic DNA was extracted from the buffy coat following the Gentra Puregene DNA Purification Protocol (Qiagen, Valencia, CA, United States). Reduced representation bisulfite sequencing (RRBS) was used to determine DNA methylation fractions using the Ovation RRBS Methyl-Seq kit (Tecan Genomics, Redwood City, CA, United States). This method uses bisulfite-treated DNA and MspI restriction enzyme, which digests the DNA into short fragments, providing a quantitative method to define and compare DNAm profiles on a genomic level (32). RRBS captures up to 80% of CpG islands and majority of promoter regions with a good coverage depth (33).

Details of the sequencing and bioinformatic methods have been previously published (24). Briefly, RRBS was performed in duplicate on five samples to determine the repeatability of the methodology. Raw reads were subjected to quality control using *FastQC* software (Babraham bioinformatics, UK) (34). *Trim Galore* was used to remove low quality reads, adapters, and RRBS related residues (35). Next, trimmed reads were aligned and mapped to the pre-indexed and bisulfite converted reference genome (hg38) with Bismark using the default parameters and Bowtie2 (36). After normalization, we counted differentially methylated cytosine (DMCs) using Bismark's methylation extractor. DNA methylation values were calculated for each DNA loci as a fraction (percent) of methylated cytosine relative to total cytosines.

## Data analysis

All phenotypic and demographic data were analyzed using SPSS for windows v27.0 (SPSS Inc., Chicago, IL, United States). Data were checked for normality, outliers, and missing values. No outliers were identified and only samples with complete phenotype and DNAm data were included in the analysis. Means and standard deviation (SD) were calculated for continuous variables, while percent (%) were used for categorical variables. Internalized stigma group differences in demographic and clinical variables were determined using the independent sample *t*-test and *χ*^2^ test based on level of measurement. Pearson correlation was conducted to examine the correlation between internalized stigma and perceived stress. Significance was set at *p < 0.05* (two-tailed).

### Differential methylation detection and gene set enrichment analyses

Differential methylation analyses were performed with the *methylKit* R package using the *calculateDiffMeth* function (37). This function uses logistic regression models to detect DMCs between low vs. high stigma. We included age, sex, and race as covariates in the models and adjusted for multiple testing using the SLIM method, obtaining *q*-values. The *q*-values are the equivalence of false discovery rate (FDR) in *methylKit*. Consistent with prior work, we defined differentially methylated loci (DML) as DMCs with *q < 0.01* and at least 10% methylation difference between groups (23, 24, 38).

The DMCs sites were mapped to hg38. Genes, gene features, and gene locations identified using *annotatr* R package (39). Genic features annotations included 1–5 Kb upstream of the transcription start sites (TSS), the promoter (<1 Kb upstream of the TSS), exons, introns, and intergenic regions. After removing duplicates, we performed annotated gene set enrichments of GO, and functional pathways using *gprofiler2* (40). Additionally, we examined the interaction between the Kyoto Encyclopedia of Genes and Genomes (KEGG) functional pathways using web-based *NetworkAnalyst 3.0* (41). For GO and functional enrichment pathway analyses, significance ware set at an adjusted *p < 0.05* (two-tailed).

## Results

### Sample characteristics

The study sample was 48 adults diagnosed with non-specific cLBP with a mean age of 44.69 year (SD = 12.92). The self-identified sex and racial distribution were approximately equal, including 27 women (56.3%) and 26 White Americans (54.2%). On average, the participants reported chronic pain intensity and disability scores of 6.4 (SD = 1.89) and 5.47 (SD = 2.67) on the GCPS, respectively. Table 1 provides demographics and a description of the variables evaluated.

**Table 1:** Demographic variables considered in analyses.

	All (*n* = 48)	Low Stigma (*n* = 30)	High Stigma (*n* = 18)	*p*-value
Pain intensity–Mean (SD)	6.40 (1.89)	5.62 (1.71)	7.51 (1.50)	<0.001
Pain disability–Mean (SD)	5.47 (2.67)	4.51 (2.52)	7.22 (2.20)	0.001
Average PSS–Mean (SD)	18.39 (6.63)	16.69 (6.55)	21.53 (5.62)	0.01
Average ICPS–Mean (SD)	1.80 (0.54)	1.43 (0.22)	2.44 (0.31)	<0.001
Age–Mean (SD) years	44.69 (12.93)	42.8 (13.42)	47.06 (11.74)	0.27
Sex–*N* (%)				
Male	21 (43.8)	11 (36.7)	11 (61.1)	0.14^a^
Female	27 (56.3)	19 (63.3)	7 (38.9)	
Race–*N* (%)				
Black	22 (45.8)	12 (40)	8 (44.4)	
White	26 (54.2)	17 (56.7)	9 (50)	0.87^a^

SD, standard deviation; PSS, perceived stress scale; ICPS, Internalized chronic pain stigma.

^a^ Denotes distribution of variable between low and high stigma compared using the *χ*^2^ tests. We determined means group differences with the independent *t*-test.

### Correlation between perceived stress and internalized stigma

**Figure 1:**
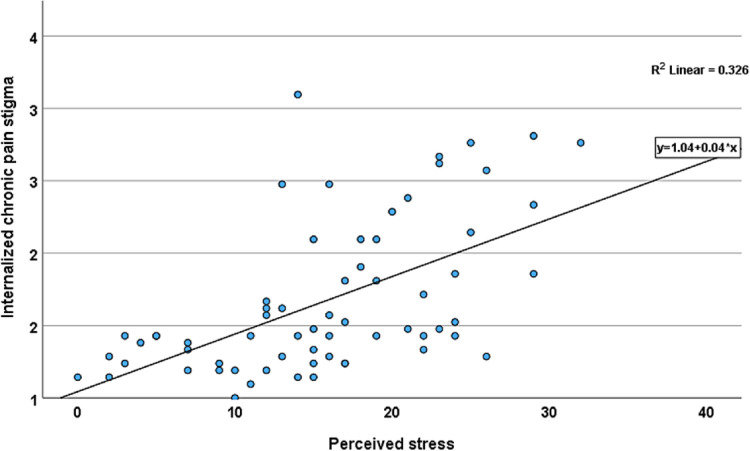
Scatter plot of the relationship between internalized chronic pain stigma and perceived stress among adults with non-specific cLBP. Line shows the linear relationship between internalized chronic pain stigma and perceived stress.

To establish that internalized stigma is a stressful experience, we determined the correlations between internalized stigma and perceived stress. There was a moderate to strong positive relationship between PSS and internalized stigma (*r* = 0.57, 95% CI [0.38–0.71] (Figure 1). Individual scores on the PSS ranged from 3 to 32, with an average score of 18.39 (SD = 6.63), and ICPS scores ranged from 1 to 3, with a mean score of 1.8 (SD = 0.54). Over half (*n* = 30, 62.5%) of the participants reported low stigma. There were no differences in age, sex, and race between adults with low and high stigma. On average, participants with high stigma reported higher pain intensity, disability, and perceived stress levels than those with low stigma (Table 1).

### Differential methylation of low and high stigma

**Figure 2:**
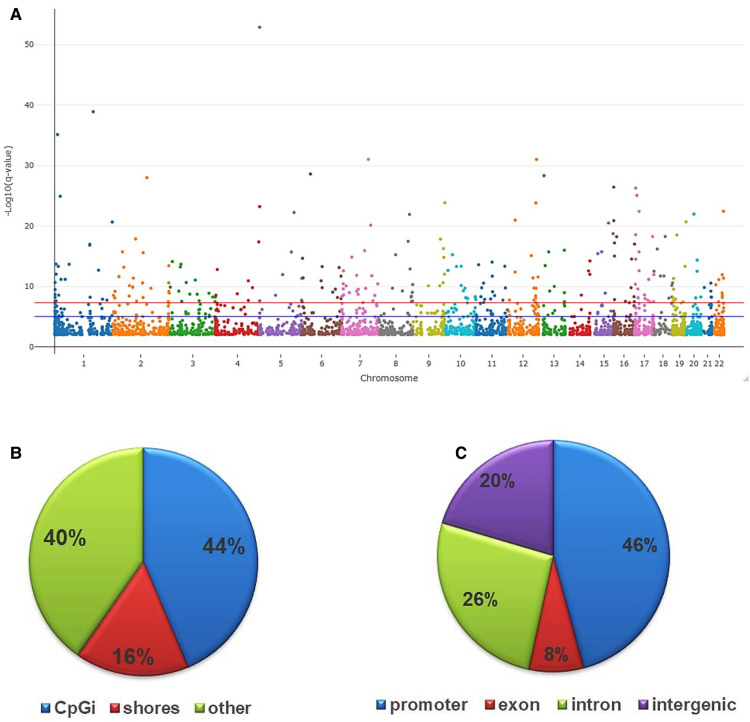
Genomic distribution of differentially methylated CpGs in low vs. high stigma in adults with non-specific cLBP. (**A**) The Manhattan plot dispicting the distribution of the CpGs on the autosome. The red line indicates the genome wide significant threshold −log10(5 × 10^−08^); blue line is the suggestive line. (**B**) Fraction of CpGs differentially methylated relative to CpG distribution on the reference genome: CpG island (44%), CpG shores (16%) and others (40%). (**C**) Fraction of CpGs differentially methylated based on their distribution on various annotated genes: promoter (46%), introns (26%), exons (8%) and intergenic regions (20%). All depicted CpGs were significantly differentially methylated between individuals with low vs. high stigma (*q* < 0.01).

As an initial step in determining DNAm profiles associated with internalized stigma, we compared the global quality of the RRBS reads for about two million CpG sites uniquely mapped to each participant's reference genomes. The mapping efficiency for the samples ranged from 62.2 to 68.8%. After controlling for age, sex, race, and multiple testing, there were 3,665 CpG sites (2,280 hypomethylated and 1,385 hypermethylated) that differed between low and high stigma adults with non-specific cLBP (*q* < 0.01). As shown in Figure 2, the differentially methylated CpGs were distributed across the 22 autosomes. The majority annotated to CpG islands (43.66%) and the promoter regions (46%) of the annotated genes.

**Figure 3:**
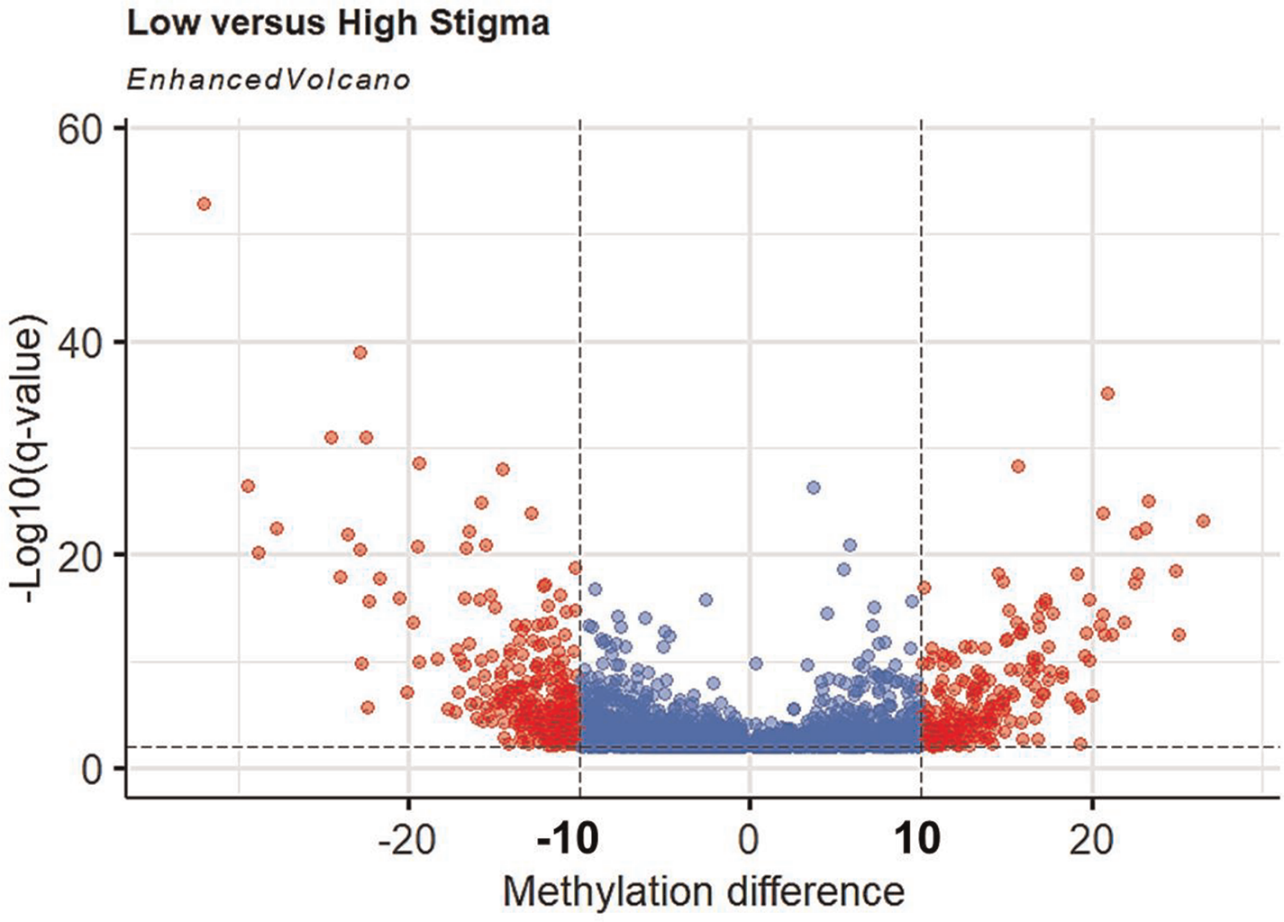
Volcano plot of hypermethylated and hypomethylated CpGs in high stigma compared to low stigma in adults with non-specific cLBP. The magnitude of the statistical significance between methylation differences are displayed as positive or negative (*x*-axis) with low stigma as reference point vs. high stigma, against −log10(*q*-value) on the *y*-axis. Black horizontal dashed line indicated a *q*-value threshold of 0.01 and vertical Black line indicates methylation difference thresholds of 10% to −10%. Red dots are DMLs (*q* < 0.01 and methylation difference ≥10).

To increase the chances of non-random distribution of methylation differences, we increased the stringency for inclusion of CpGs as a DML using *q* < 0.01 and ≥ 10% methylation difference between groups. With the additional criteria, we identified 527 DML in individuals with high stigma compared to low stigma. Among the DML, methylation was decreased in 289 loci and increased in 238 loci in individuals with high stigma. Figure 3 depicts the magnitude of methylation differences between low and high stigma individuals. Table 2 summarizes genomic loci and associated methylation differences of the top 20 DML between low vs. high internalized stigma among adults with non-specific cLBP. These include genes that have previously been associated with pain, such as *SSTR5* (42), *FOXP2* (43, 44), and *IL22RA1* (45).

**Table 2:** Top 20 differentially methylated loci between low vs. high stigma.

Chr	Position	*p*-value	*q*-value	Methylation difference	Genes	Gene name/description
5	163,653	3.32 × 10^−59^	1.26 × 10^−53^	−31.99	LOC107986394	ncRNA
1	165,367,963	6.45 × 10^−45^	1.23 × 10^−39^	−22.86	LMX1A	LIM Homeobox Transcription Factor 1 Alpha
1	12,666,306	5.86 × 10^−41^	7.43 × 10^−36^	20.95	AADACL4	Arylacetamide Deacetylase Like 4
12	124,301,507	1.22 × 10^−36^	9.53 × 10^−32^	−24.53	RFLNA	Refilin A
7	114,384,123	1.25 × 10^−36^	9.53 × 10^−32^	−22.49	FOXP2	Forkhead box protein P2
6	36,509,901	3.91 × 10^−34^	2.48 × 10^−29^	−19.39	STK38	Serine/Threonine Kinase 38
13	23,142,926	8.78 × 10^−34^	4.77 × 10^−29^	15.67	LOC105370112	ncRNA
2	146,232,211	2.09 × 10^−33^	9.94 × 10^−29^	−14.53	LOC105373667	ncRNA
16	1,079,872	8.82 × 10^−32^	3.73 × 10^−27^	−29.42	SSTR5	somatostatin receptor 5
17	5,062,549	1.41 × 10^−31^	5.37 × 10^−27^	3.71	ZFP3	Zinc finger protein 3
17	10,106,299	2.53 × 10^−30^	8.73 × 10^−26^	23.36	GAS7	Growth arrest specific 7
1	24,148,032	3.76 × 10^−30^	1.19 × 10^−25^	−15.75	IL22RA1	Interleukin 22 receptor subunit alpha 1
9	137,729,700	4.91 × 10^−29^	1.44 × 10^−24^	20.67	EHMT1	Euchromatic histone lysine methyltransferase 1
12	121,356,310	5.60 × 10^−29^	1.52 × 10^−24^	−12.85	ANAPC5	Anaphase promoting complex subunit 5
4	189,266,846	2.44 × 10^−28^	6.17 × 10^−24^	26.49	LOC105377614	ncRNA
22	49,659,272	1.56 × 10^−27^	3.71 × 10^−23^	−27.79	C22orf34	ncRNA
17	19,506,661	1.75 × 10^−27^	3.91 × 10^−23^	23.15	LOC112268205	ncRNA
5	147,296,454	2.72 × 10^−27^	5.75 × 10^−23^	−16.47	STK32A	Serine/threonine kinase 32A
20	32,984,768	5.42 × 10^−27^	1.09 × 10^−22^	22.64	SUN5	Sad1 and UNC84 domain containing 5
8	131,465,165	6.46 × 10^−27^	1.23 × 10^−22^	−23.58	LOC105375763	ncRNA

Chr, chromosome; ncRNA, non-coding ribonucleic acid; Comparison of the fraction of methylated cytosine across the low vs. high stigma group. *p*-values were calculated with the methylkit logistic regression, and the sliding linear model (SLIM) method was used to correct *p*-values to *q*-values.

### Gene ontologies enriched by DMLs in low and high stigma in non-specific cLBP

**Figure 4:**
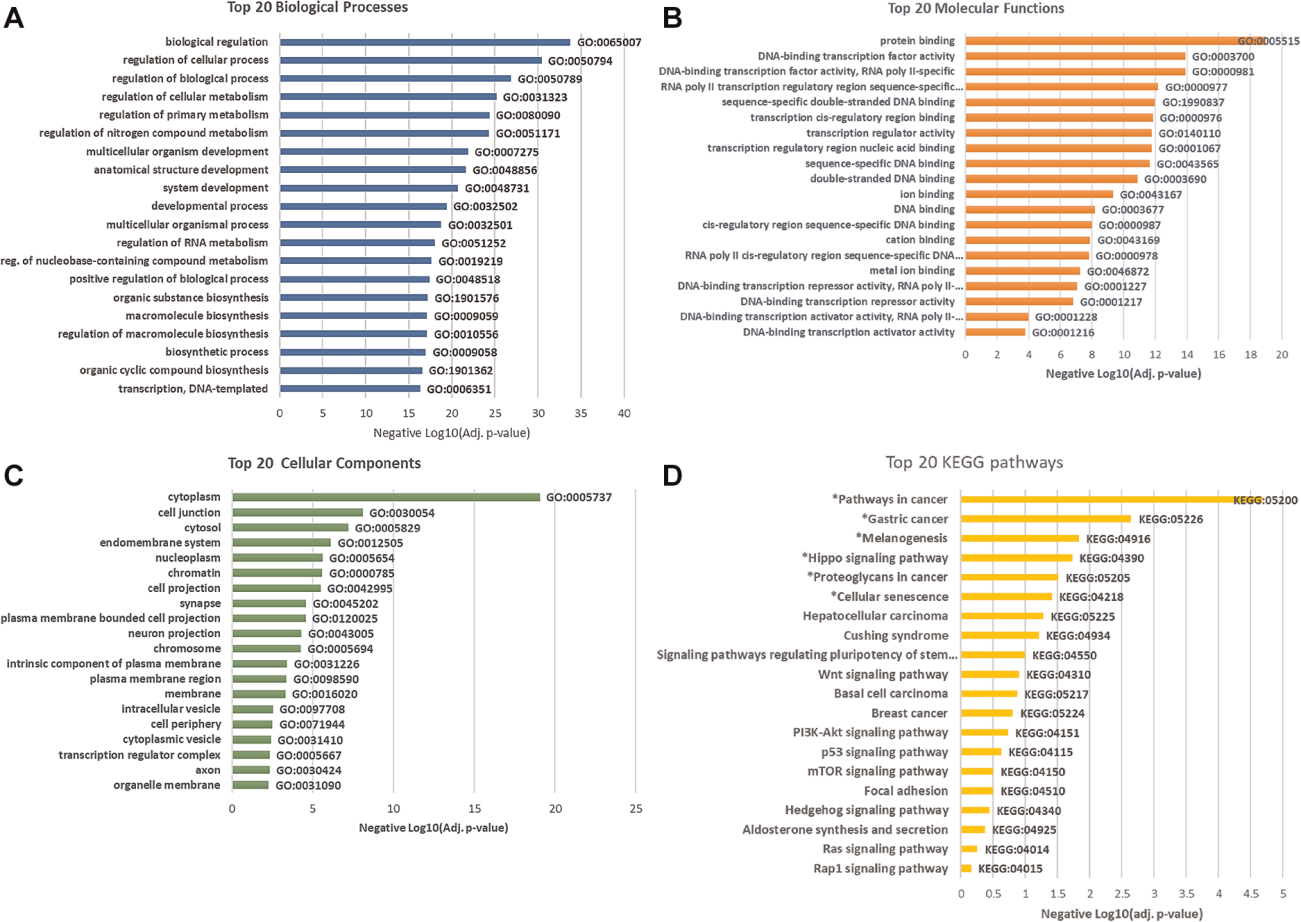
Go and KEGG pathway enrichment by differentially methylated gene in low vs. high stigma showing biologically processes (**A**) Molecular functions (**B**) Cellular components (**C**) and KEGG pathways (**D**). All depicted GO were significant and *denotes significant KEGG pathways (adj. *p* < 0.05).

After removing duplicates, GO analyses revealed significant enrichment of 327 terms (adjusted *p* < 0.05): 274 biological processes (BP), 29 cellular components (CC), and 24 molecular functions (MF). Many of the top enriched GO termed have important regulatory functions, including the regulation of cellular processes (GO:0050794, adj. *p* = 2.51 × 10^−30^), biological regulation (GO:0065007; adj. *p* = 7.31 × 10^−34^), protein binding (GO:0005515; adj. *p* = 5.03 × 10^−21^), and regulation of RNA metabolic processes (GO:0051252; adj. *p* = 2.28 × 10^−18^). Figures 4A–C depict the top 20 biological processes, cellular components, and molecular functions in low vs. high stigma groups.

### Pathway analysis of DMLs-associated genes in low and high stigma in non-specific cLBP

To identify and understand functionally related groups of genes coordinately affected by the differential methylation between low and high stigma, we entered the unique gene names into the KEGG and NetworkAnalyst Databases. The top pathways significantly enriched by genes whose methylation levels were different between low vs. high stigma included Pathways in cancer (89 genes, adj. *p* = 6.77 × 10^−5^), Gastric cancer (32 genes, adj. *p* = 0.002), Melanogenesis (23genes, adj. *p* = 0.01), Hippo signaling (31 genes, adj. *p* = 0.017), proteoglycans in cancer (37 genes, adj. *p* = 0.03): and cellular senescence (30 genes, adj. *p* = 0.04). Figure 4D depicts the top 20 KEGG pathways over-represented by the differentially methylated genes.

**Figure 5:**
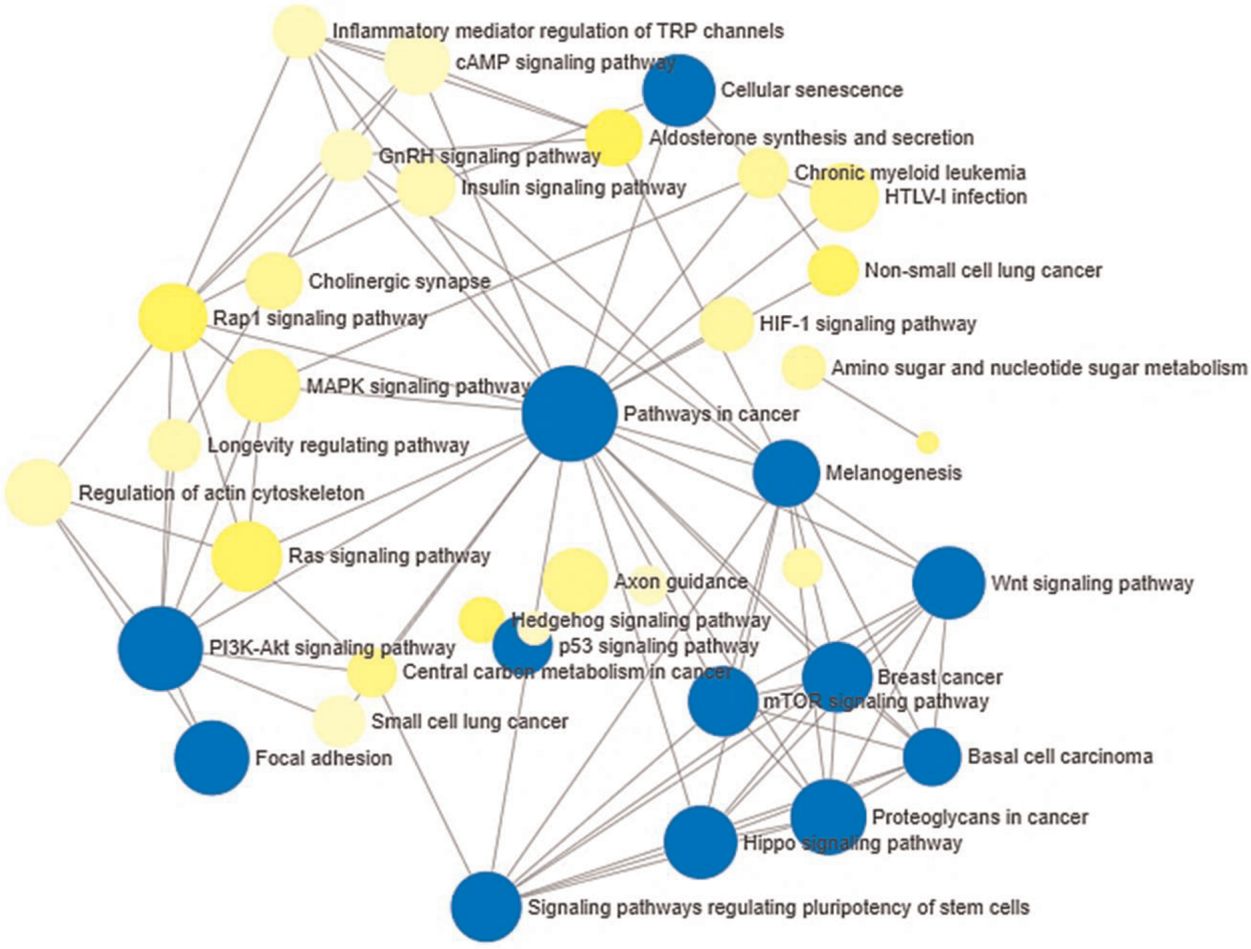
Network analysis of KEGG pathways enriched by annotated differentially methylated (hypo- and hypermethylated) gene in low vs. high stigma. Node sizes are proportional to the number of differentially methylated genes. Blue node = adj. *p* < 0.05 using the hypergeometric tests in Network Analyst.

We also examined the interaction between the KEGG pathways using network analyses. The network analyses revealed significant interactions between many pathways of relevance to stress and neuroinflammatory processes, including Hippo Signaling, P13K-Akt signaling, p53 signaling, mTOR signaling, and Wnt signaling pathways (Figure 5).

## Discussion

Non-specific cLBP is challenging to diagnose and manage, leading to internalized stigma and many years lived with disability (1, 2, 19). Internalized stigma has been associated with insomnia, depressive symptoms, catastrophizing, and overall poorer outcomes (10, 13). To date, most studies of internalized stigma in cLBP individuals have focused on mediating and moderating roles of psychosocial factors (7, 11–13, 19). To our knowledge, this represents the first study correlating internalized stigma with DNAm profiles in individuals with non-specific cLBP. We report stigma-associated DNAm differences at numerous CpG sites that annotated to genes of relevance to stress and pain pathology such as *SSTR5* (42), *FOXP2* (43, 44), and *IL22RA1* (45). Given the high number of differentially methylated genes, we will focus this discussion on the molecular functions, enrichment pathways, and network interaction over-represented by these differentially methylated annotated genes. We observed an over-representation of genes involved in regulatory processes (e.g., DNA-binding transcription factor activity, protein binding, and transcription regulatory activity) and neuroinflammatory pathways (e.g., pathways involved in *Hippo signaling*, *mTOR signaling*, *Wnt Signaling,* and *PI3K-Akt signaling*). These findings are consistent with previous studies suggesting a relationship between pain and stress-related molecular changes in the expression of neuroinflammatory genes (46–48).

*Hippo signaling* is an evolutionary conserved network that plays a critical role by regulating many biological mechanisms and functions, including cell proliferation, apoptosis, organ size, and organ regeneration (49). Similarly, *Wnt signaling* is essential for organogenesis, neuronal and stem cell regeneration, synaptic formation, and neuroplasticity in adults (50). Differential expression of genes in *Hippo* and *Wnt signaling* pathways have been associated with chronic pain conditions (50, 51). We identified significant overrepresentation of the *Hippo Signaling pathway* by genes differentially methylated between adults with low vs. high internalized stigma. Additionally, our network analysis confirmed a link between *Hippo* and *Wnt signaling pathways*. Our findings are consistent with existing literature supporting crosstalk between *Hippo* and *Wnt Signaling pathways* in various stress-related, chronic pain, and neurological conditions. *Hippo* and *Wnt signaling* pathways integrate several biological processes through downstream effectors (e.g., *β*-catenin) and post-transcriptional modification of various mediators such as TGF-*β* (52, 53). Hippo signaling is regulated by mechanical stress, contact inhibition, and sheer force (54). Dysregulation of *Hippo* and *Wnt signaling* pathways are associated with various cancers and multiple neurodevelopmental (52), metabolic, neurodegenerative (52), and neuroinflammatory (53) diseases. Previously, we associated differential methylation of genes in *Hippo signaling* pathways with non-specific cLBP (24). Other investigators have reported the potential role of *Hippo and Wnt signaling* in stress-related neuropsychiatric disorders, including depressive symptoms and post-traumatic stress disorder, which are known predictors of worse pain outcomes (55, 56). Also, increased glucocorticoid receptor activity has been associated with increased fibronectin expression and subsequently enhanced Hippo signaling, which increases hypothalamic-pituitary-adrenal axis activity, perhaps through post-transcription modifications in Wnt pathways (56). Thus, it is possible that stigma-associated chronic stress induces epigenetic modifications that affect Hippo and Wnt signaling pathways, making cLBP worse for adults with high levels of internalized stigma.

Another insight from our findings is the significant interaction between *mTOR*, *p53*, and *PI3K-Akt signaling* pathways. p53 is a well-known tumor suppressor protein that transmits cellular stress signals in response to DNA damage, hypoxia, and nucleotide deprivation, inducing cell cycle arrest or cell death. It plays an important role in maintaining genomic stability and cellular homeostasis. Emerging evidence from animal studies suggest that p53 signaling mediate the relationship between epigenetic modifications and chronic pain (57). Decreased expression of the p*53* gene can reduce p53 protein levels and alleviate neuropathic pain (58). Other investigators have suggested that inhibition of p53 signaling also decreases the inhibition of inflammatory cytokines in neuropathic pain (59). Similarly, recent studies suggest that elevated levels of neuroendocrine hormones, including cortisol and cortisone (a major glucocorticoid in mice) down-regulate p53 protein levels and signaling (60). Also, chronic stress and associated elevated glucocorticoid levels suppress p53 functions (60, 61). Thus, differential methylation of genes involved in the p53 signaling pathway may play a role in the relationship between chronic pain and chronic stress.

The PI3K-Akt/mTOR signaling pathway regulates many key processes, including inflammatory response, cellular growth, and cellular apoptosis, in response to various cues, including oxidative stress, DNA damage, and nutrients (62, 63). This pathway contains three lipid kinases, P13K (phosphoinositide 3-kinase), Akt (As protein kinase B), and mTOR (mammalian target of rapamycin), which maintain homeostasis by mediating inflammatory cytokines (63), and regulating cartilage repair (62). Inhibition of the PI3K-Akt/mTOR pathway decreases inflammatory response in rats with osteoarthritis (62), and genetic mutations in the pathways have been associated with susceptibility to knee osteoarthritis in humans (64). Other investigators have reported that epigenetic activation of PI3K-Akt/mTOR pathway may protect against intervertebral degenerative disc disease (65, 66). Epigenetic modification of targets in these pathways may reduce neuroinflammation (67) and relieve intervertebral disc degeneration (68). However, the exact role of the PI3K-Akt/mTOR pathway mediated by DNAm changes in non-specific cLBP or associated internalized stigma is still unclear and warrants further research. In this study, we found that these pathways were enriched by differentially methylated genes between adults with non-specific cLBP who report low vs. high internalized stigma. The network analysis showed significant interaction between these pathways, suggesting that the PI3K-Akt/mTOR pathways might be functionally involved in the pathogenesis of non-specific cLBP and the high pain severity and disability associated with higher internalized stigma. Given that the PI3K-Akt/mTOR pathway plays a key role in delaying inflammation, it is possible that stress of internalized stigma affects non-specific cLBP through epigenetically induced dysregulation of inflammatory response.

Despite being the first study investigating epigenetic changes and internalized stigma in adults with non-specific cLBP, the present study has some limitations. First, causality cannot be determined because our study was cross-sectional in nature. Second, epigenetic modifications are cell-type specific, and this study was based on whole blood samples and not specific tissues in the nervous system related to pain processing such as the dorsal root ganglion. We identified differentially methylated genes and pathways with neuroinflammatory and chondrocyte repair functions that might contribute to worse non-specific cLBP for individuals with high stigma. Nevertheless, the use of blood samples increases the potential clinical useability of the findings. Finally, our sample size was relatively small. However, Tsai et al. (69) have previously observed that a sample of 37 provides more than 80% power to detect an 7% mean methylation difference in a case-control design at *p* < 0.05. Clearly, our sample size of 48 was sufficient to detect a 10% methylation difference between low vs. high chronic pain stigma.

In conclusion, there is a strong correlation between internalized stigma and pain outcomes. Higher levels of internalized stigma correlate with poorer wellbeing, greater depression, greater pain intensity and disability among adults with non-specific cLBP. Our findings suggest that differential DNAm of genes are overrepresented in pathways involved in neuroinflammation, neurodegeneration, and stress. These findings provide the first proof-of-concept data suggesting that DNA methylation differences in stress and immune regulatory pathways may explain the relationship between internalized stigma and pain outcomes in adults with non-specific cLBP. Interventions to reduce internalized stigma and reverse these epigenetic changes may improve non-specific cLBP outcomes.

## Data Availability

Publicly available datasets were analyzed in this study. This data can be found here: ncbi.nlm.nih.gov, accession number: PRJNA894531.
